# Clinicopathological findings in a case series of abdominopelvic solitary fibrous tumors

**DOI:** 10.3892/ol.2014.1872

**Published:** 2014-02-11

**Authors:** HAO WANG, PING CHEN, WEI ZHAO, LEI SHI, XUEWEN GU, QING XU

**Affiliations:** 1 Department of Gastrointestinal Surgery, Northern Jiangsu People’s Hospital, Yangzhou University, Yangzhou, Jiangsu 225001, P.R. China; 2 Department of Pathology, Northern Jiangsu People’s Hospital, Yangzhou University, Yangzhou, Jiangsu 225001, P.R. China

**Keywords:** solitary fibrous tumor, spindle cell tumor, histopathology, immunohistochemistry

## Abstract

Solitary fibrous tumors (SFTs) represent a rare type of soft tissue tumor. Extrathoracic SFTs (ESFTs) in the soft tissues of the abdominopelvic cavity are extremely rare. Between January 2002 and January 2013, 10 patients were identified with abdominopelvic SFTs at the Northern Jiangsu People’s Hospital. The clinicopathological data, treatment and follow-up results were retrospectively analyzed in this study. Patients included four females and six males, whose age ranged between 21 and 75 years (mean, 53.3 years). The maximum diameter of the tumors was 2.5–28 cm (mean, 12.7 cm). Two cases were diagnosed as malignant variants of ESFTs. R_0_ resection was performed in eight patients, while one patient underwent R_1_ resection, and one patient received palliative chemotherapy for an inoperable mass. Follow-up time ranged between 6 and 126 months (mean, 50 months). The patient with R_1_ resection suffered a local relapse, and the patient receiving palliative chemotherapy succumbed to the disease. The remaining eight patients remained free of disease. Abdominopelvic SFTs usually reveal an indolent process, although the majority of tumors in the present study were of giant size when diagnosed. The risk of local recurrence and metastasis correlates with tumor size and the histological status of surgical margins. The preferred treatment is complete resection followed by extended follow-up surveillance.

## Introduction

Solitary fibrous tumors (SFTs), also named hemangiopericytomas, are rare spindle cell tumors first documented as arising from the pleura by Klemperer and Rabin (1). SFTs are rare entities accounting for <2% of all soft tissue sarcomas (2). Although the majority of reported tumors arise in the thoracic cavity, SFTs from a wide range of anatomic sites have been reported (3–6). Extrathoracic SFTs (ESFTs), particularly those in the abdominal and pelvic cavities, are rare among soft tissue tumors. In a more recent retrospective study, abdominopelvic SFTs accounted for 34% of all SFTs, illustrating that the abdominopelvic cavity has become the major primary site of SFTs (7). Patients with abdominopelvic SFTs may present with abdominal distention/pain, a palpable mass and neurological or vascular symptoms. Hypoglycemia may also be observed in certain cases. However the association between clinical behavior and histopathological characteristics of abdominopelvic SFTs requires further clarification. Usually, the tumor follows an indolent clinical course with no recurrence and metastasis, yet its elusive clinical behavior makes it impossible to provide an exact prognostic prediction and between benign and malignant SFTs. In the present study, 10 cases of abdominopelvic SFTs were retrospectively analyzed to highlight the clinicopathological profiles of this rare entity.

## Patients and methods

### Patient identification

Between January, 2002 and January, 2013, 10 abdominopelvic tumors were histologically identified as SFTs at the Northern Jiangsu People’s Hospital (Yangzhou, China). Clinical data were collected from discharge records, operating theater archives and telephone calls to the patients. Follow-up data were available for all patients and consisted of clinical examinations, chest X-rays, abdominal ultrasounds and computed tomography (CT) or positron emission tomography-computed tomography (PET) of the tumor site. This retrospective study was approved by the ethics committee of the Northern Jiangsu People’s Hospital. The patients consented to the publication of this study.

### Pathological review

Fine needle aspiration biopsy specimens were obtained in one case. The resection specimens were evaluated for tumor size, primary location, surgical margin and cut surface. A macroscopic photograph of the cut surface was obtained in selected cases. Histopathological examination was performed by two experienced soft tissue tumor pathologists (Xuewen Gu and Qing Xu). The diagnosis was confirmed by morphological and immunohistochemical (IHC) findings available for review. The pathological diagnostic criteria of SFT used were circumscribed tumors characterized by a haphazard growth pattern (‘patternless pattern’) of short spindle cells with scant cytoplasm and bland cytological appearance separated by strands of rope-like collagen. IHC analysis included the following antibodies, supplied by Zhongshan Golden Bridge Biotechnology, Inc. (Beijing, China): Cluster of differentiation (CD)34, Bcl-2, CD99, CD117, cytokeratin (CK) pan, epithelial membrane antigen (EMA), S-100 protein, vimentin, smooth muscle actin (SMA), desmin, Ki-67, insulin receptor [IR; sc-20739, Santa Cruz Biotechnology, Inc. (Santa Cruz, CA, USA)] and insulin-like growth factor 1 receptor (IGF-1R). Tumors were scored for Ki-67 labeling index, mitotic activity [mitotic fields per 10 high-power fields (HPFs)], cellularity, nuclear pleomorphism and necrosis. The identification of a malignant component was based on the mitotic count (activity in ≥4/10 high-power fields) and the presence of necrosis and nuclear pleomorphism.

## Results

### Clincal features

The present cohort of patients included six males and four females, with a mean age at presentation of 53.3 years (range, 21–75 years). The tumors existed 6 months to 10 years prior to diagnosis. The clinical features of the 10 cases are summarized in Table I. Tumors remained painless or became symptomatic by their mass effect, causing localized pain, distension, or, as in one patient (no. 5), constipation. Hypoglycemia was observed only in one patient (no. 1). There were no other symptoms. In one patient (no. 2), the mass remained asymptomatic and was identified incidentally in a routine physical examination. One patient (no. 1) was admitted to the Northern Jiangsu People’s Hospital for emergency surgery due to spontaneous tumor rupture and hypovolemic shock.

### Radiological findings

**Figure 1:**
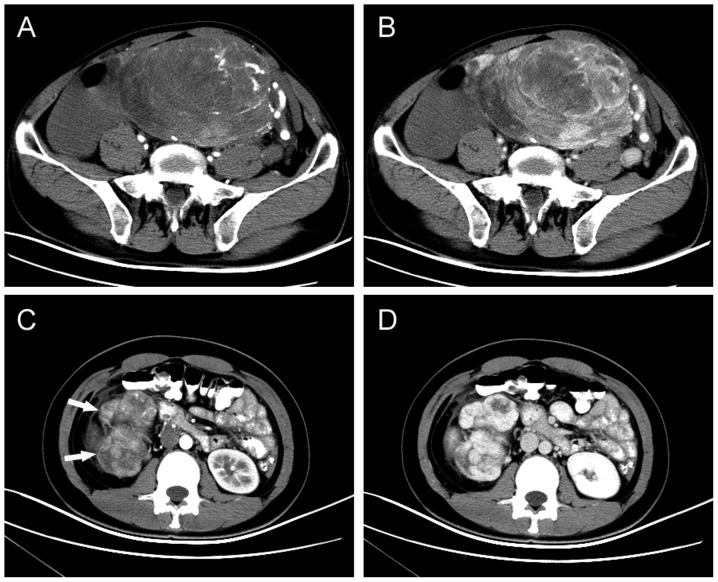
Typical imaging manifestations of abdominopelvic SFTs. (A) Arterial-phase and (B) venous-phase axial contrast-enhanced CT scan showing a well-defined, intensely enhancing mass in the pelvis with central nonenhancing areas. (C) Lobulated SFTs (indicated by arrows) of the retroperitoneum in a 21-year-old female. (D) Axial contrast-enhanced CT scan, obtained in the arterial phase, revealing a well-defined hypervascular mass with intense enhancement. SFT, solitary fibrous tumor; CT, computed tomography.

**Figure 2:**
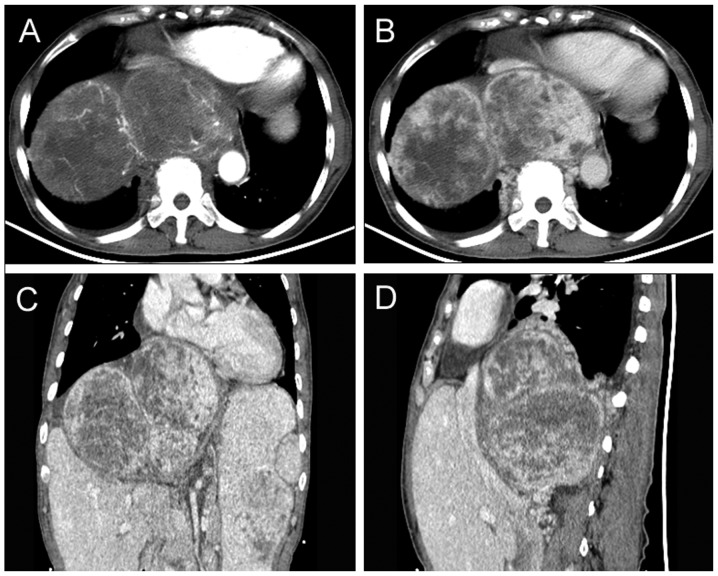
(A) Arterial-phase CT scan. The tumor shows moderate to marked enhancement, with the tumor edge visible for tortuous vascular shadow. (B) Venous-phase CT scan demonstrating sustained enhancement of the tumor. Nonenhanced areas suggest central cystic degeneration and necrosis. (C) Coronal and (D) sagittal reconstructed images: The tumor is lobulated, comprised of three masses. The heart, liver and right kidney were pressured significantly due to the tumor mass effect. CT, computed tomography.

All patients received CT scans prior to diagnosis. Of the 10 tumors, three were located in the retroperitoneum, two in the sigmoid mesocolon, three in the pelvis, and one each in the greater omentum and the small bowel mesentery. The maximum diameter of the tumors was 2.5–28 cm (mean, 12.7 cm). SFTs appeared as well-circumscribed hypervascular masses (two were lobulated and eight were round) that displaced or exerted pressure effects on neighboring organs, including the liver, bowel, vessels, kidneys, bladder and ureter. Central hypoenhancing or nonenhancing areas could be observed in the tumors, which represent necrosis, cystic change or hemorrhage (Figs. 1 and 2). PET/CT examination was performed in one patient who underwent R_1_ resection and suffered local relapse. The recurrent mass, located between the bladder and rectum, showed heterogeneous uptake of fluorodeoxyglucose, and the initial standardized uptake value, normalized to lean body mass, was 5.64.

### Management

None of the patients had a history of benign or malignant tumors. In eight patients, primary resection was performed with negative surgical margins. One out of the 10 patients (no. 5) received R_1_ resection and adjuvant chemotherapy, as the tumor was located between the bladder and the rectum and had adhered to the right seminal vesicle. One patient (no. 2) with a giant, inoperable tumor located between the liver and diaphragm received palliative chemotherapy.

### Histological features

**Figure 3:**
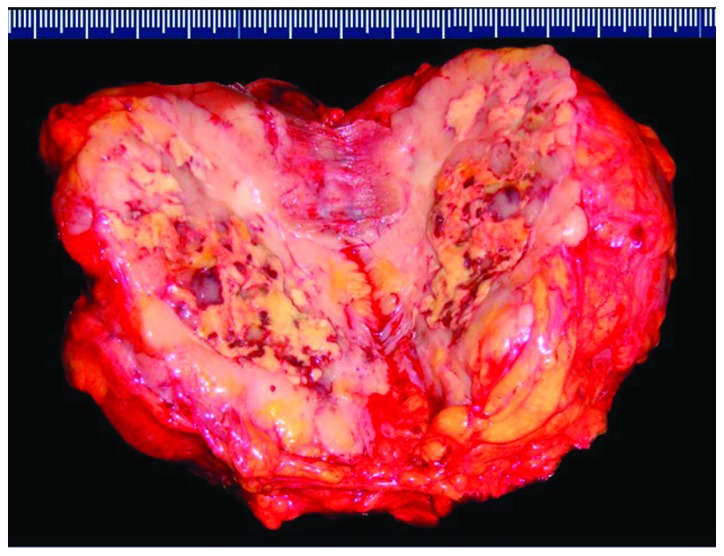
Macroscopic appearance of an atypical malignant solitary fibrous tumor (patient no. 5). The cut surface of a well-circumscribed tumor, white- to tan-colored with deeply yellow necrotic areas.

**Figure 4:**
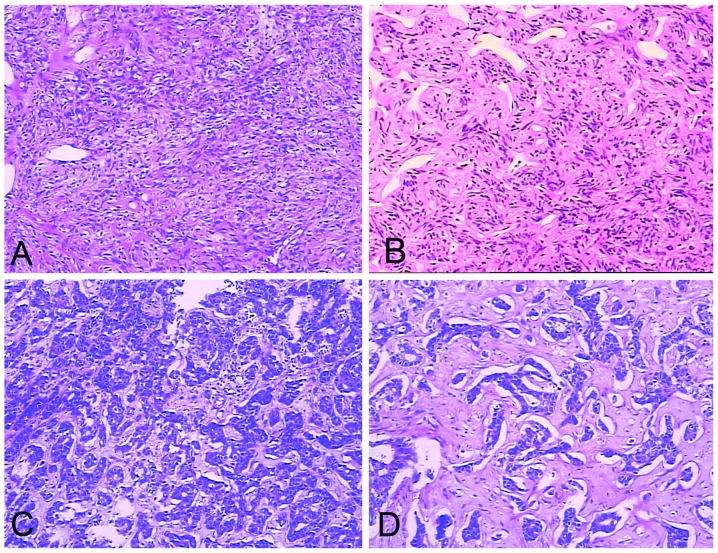
Hematoxylin and eosin stained sections. (A) SFT consists of tightly packed round to fusiform cells with indistinct cytoplasmic borders which are arranged around a vessel. Dimensions, 10×10 cm. (B) The vessels formed a continuous, ramifying vascular network. The vessels divide and communicate with small or minute vessels which have been partly compressed and obscured by the surrounding cellular proliferation. Typically, the dividing sinusoidal vessels have an ‘antler-like’ configuration. Dimensions, 10×10 cm. (C) Malignant SFT with heightened cellularity. The tumor also demonstrates marked pleomorphism with a high level of mitotic activity (>4 mitotic fields/10 high power fields). Dimensions, 10×10 cm. (D) SFT tumor cells arranged randomly in a ‘patternless pattern’. The SFT presented marked hyalinization and had characteristic artifactual ‘cracks’ between the cells and collagen. Dimensions, 10×20 cm. SFT, solitary fibrous tumor.

A CT-guided fine needle aspiration biopsy specimen was obtained in one patient (no. 2), while resected specimens were obtained in the remaining nine patients. The tumors appeared as solid, well-encapsulated and smooth to firm or soft tissue masses, and had a gray-white to red-brown color on the cut surface (Fig. 3). The tumors consisted primarily of spindle cells. The arrangement of the cells varied in different areas of the tumors. In certain areas the cells were arranged in short, ill-defined fascicles, whereas in other areas, cells were arranged at random in a ‘patternless pattern’. The tumor matrix included variable amounts of partly hyalinzed collagen bundles and hyalinization was observed in certain areas. Artifactual ‘cracks’ between the cells and collagen were observed (Fig. 4). The mitotic rate in morphologically benign SFTs was <4 mitotic fields/10 HPFs. Two lesions were diagnosed as atypical or malignant variants of ESFTs due to markedly increased cellularity, cellular atypia (nuclear pleomorphism, nuclear hyperchromasia), increased mitotic index and tumor necrosis (patient nos. 1 and 5).

### IHC analysis

**Figure 5:**
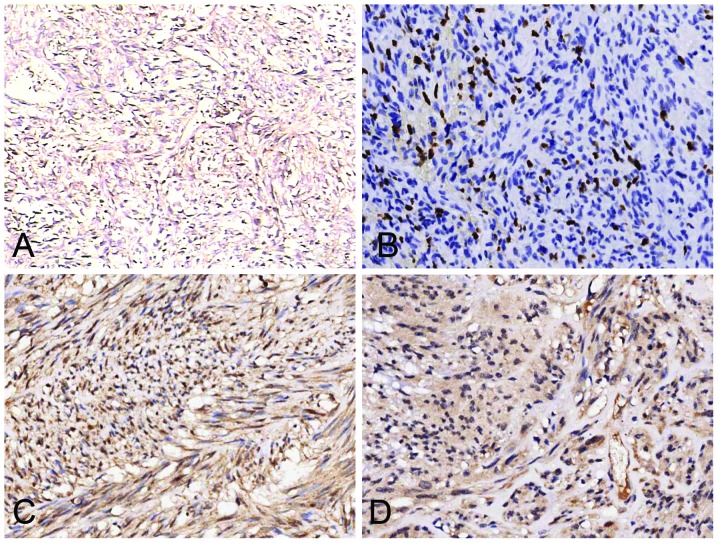
Immunohistochemical staining. (A) The majority of tumors are diffusely positive for cluster of differentiation 34. Dimensions, 10×10 cm. (B) The Ki-67 labeling index is >5% in the solitary fibrous tumor identified as a malignant variant (patient no. 1). Dimensions, 10×20 cm. Tumors were positive for (C) insulin receptor in patient nos. 1, 2 and 4–8 and (D) insulin-like growth factor 1 receptor in patient nos. 1 and 4. Dimensions, 10×20 cm.

ESFTs of abdominopelvic origin commonly expressed CD34 (90%), vimentin (70%), CD99 (60%) and Bcl-2 (50%), and less commonly expressed SMA (40%), EMA (20%) and S-100 (10%). CD117, CK pan and desmin were absent. In addition, special attention was paid to the expression pattern of IR and IGF-1R (Fig. 5). A detailed summary of the histopathological findings is provided in Table II.

### Follow-up

Follow-up data were available for all patients and consisted of clinical examination including chest X-ray, abdominal ultrasonographic examination and CT or PET/CT of the tumor site. Follow-up time ranged between 6 and 126 months (mean, 50 months). Patient statuses at last follow-up are summarized in Table I.

## Discussion

SFTs, first reported by Klemperer and Rabin in 1931 (1), are rare mesenchymal neoplasms that account for <2% of all soft tissue tumors (2). Although SFTs were previously thought to exclusively involve the pleura, it is now established that SFTs can originate in almost any part of the body, with ESFTs being more common than pleural SFTs (3–6). In 2012, Demicco *et al* (7) conducted a retrospective study of 110 cases of thoracic and extrathoracic SFTs, and found that the majority of cases were located in the abdominopelvic cavity (34%). The pleura and extremities were less common primary sites (28% and 16%, respectively) and ~22% of cases arose in the soft tissue of the head and neck (11%) or trunk (11%). In the present study, 10 cases of SFTs in the abdomen and pelvis were analyzed retrospectively.

SFTs in the abdomen and pelvis are primarily tumors of adult life which affect both genders equally. Clinically, SFTs manifest as slow-growing, often asymptomatic masses. Common symptoms include abdominal pain, a palpable mass, and neurological or vascular symptoms. Symptoms due to mass effect, including urinary retention, bowel obstruction or constipation, and abdominal distention, may be observed with tumors in the abdomen or pelvis (8). In the present study, tumors remained asymptomatic for 6 months to 10 years, until mass effects became apparent. The tumor size of abdominopelvic SFTs is usually large (commonly >10 cm).

Hypoglycemia has been reported in ~5% of SFTs, particularly in malignant SFTs, cases of Doege-Potter syndrome and, most frequently, in tumors located in the pelvis and retroperitoneum. It is mediated through production of IGFs by the tumor. IGFs and IGF-R mRNA can be identified in tumor cells even in the absence of clinical hypoglycemia (9,10). An IHC study on three SFTs with hypoglycemia revealed marked staining for IGF-1R (11). By contrast, Li *et al* (12) demonstrated the uniform activation of the IR pathway in SFTs, but failed to identify expression of IGF-1R in these tumors. Thus, the authors suggested that IGF-2-mediated downstream signaling occurs through IR rather than IGF-1R. Similarly, Hajdu *et al* (13) reported that IGF-2 expression was consistently upregulated in SFTs of all anatomic sites, and that overexpression of IR paralleled that of IGF-2 in the majority of SFTs while IGF-1R expression was consistently negligible. By using an IHC analysis, the present study demonstrated that IR is commonly expressed (60%) in SFT tissues that were identified as malignant or moderate-risk cases, whereas IGF-1R was only expressed in two cases. The current study validates the published results (12,13) and supports the suggestion that IGF-2/IR autocrine loop activation plays an oncogenic role in SFTs.

Macroscopically, the majority of SFTs appear as rounded (occasionally lobulated), encapsulated masses of homogenous density with a yellow-brown to white whorled appearance of the cut surface. Areas of necrosis and hemorrhage can be observed in tumors of large size. Under microscopic analysis, SFTs are typically composed of juxtaposed hyper- and hypocellular spindle cell proliferation, a dense collagenous matrix, and numerous thin-walled blood vessels with an antler-like configuration (a histological hallmark of SFT) (14). Immunohistochemically, ESFTs primarily express CD34 (80–90%), CD99 (70%), Bcl-2 (30%), EMA (30%) and SMA (20%) (15–21). Desmin, CK and S-100 protein are usually absent (15). The IHC analysis results of the current study validate these findings.

The majority of SFTs are benign (~78–88%) and 12–22% are malignant (22,23). The criteria proposed by England *et al* (24) for malignant SFTs are large size (>5 cm), increased mitotic rate (≥4 mitotic fields/10 HPFs), high cellularity, pleomorphism, presence of hemorrhage and necrosis. In the present study, two out of 10 cases were identified as malignant SFT (no. 1 and no. 5). By contrast, Demicco *et al* (7) suggested using a risk stratification model (low, moderate and high risk) based on age (<55 or ≥55 years), tumor size (<5, 5–10, 10–15 and ≥15 cm) and mitotic index (0, 1–3 and ≥4 mitotic fields/10 HPFs) to predict SFT behavior (metastasis and mortality), rather than simply classifying tumors as either benign or malignant.

Surgical excision remains the treatment of choice for SFTs. All patients undergoing complete surgical excision were alive at five years following treatment (25). Surgical resectability is the most important prognostic factor (24). In the present study, the only patient to suffer a local relapse was primarily resected incompletely. Although adjuvant treatment has been introduced into cases of incompletely resected or inoperable SFTs, no significant benefits of adjuvant radiation therapy or chemotherapy have been reported. In the current study, the patient who received palliative chemotherapy due to an inoperable SFT succumbed to the disease 32 months after diagnosis, and the patient who received adjuvant chemotherapy for an incomplete resection suffered local relapse 6 months later.

SFTs may develop late recurrences or metastases even in cases which have been identified as benign. Thus, long follow-up periods (≥15 years) should be maintained with closer follow-up during the first two years (26).

In conclusion, the majority of abdominopelvic SFTs follow a benign clinical course following surgical resection with free margins. Closer surveillance is warranted for those tumors that are >10 cm or have a component of histological malignancy.

## Figures and Tables

**Table I:** Clinical features of abdominopelvic SFTs.

No.	Gender	Age, years	First manifestation	Location	Size, cm	Management	Recurrence/metastasis	Follow-up time, months	Follow-up status
1	M	49	Hypoglycemia, tumor rupture	Sigmoid mesocolon	16.9	R_0_ resection	No	13	Alive
2	F	62	Asymptomatic	Retroperitoneum	10.5	Palliative chemotherapy	No	32	Deceased
3	F	21	Painless mass	Retroperitoneum	10.3	R_0_ resection	No	21	Alive
4	M	29	Abdominal pain, distention	Greater omentum	28.0	R_0_ resection	No	60	Alive
5	M	56	Constipation	Pelvis	9.5	R_1_ + adjuvant chemotherapy	Local recurrence	6	Alive
6	M	72	Painless mass	Small bowel mesentery	17.0	R_0_ resection	No	18	Alive
7	M	40	Painless mass	Sigmoid mesocolon	12.8	R_0_ resection	No	53	Alive
8	F	61	Abdominal pain	Retroperitoneum	14.0	R_0_ resection	No	75	Alive
9	F	75	Abdominal pain	Pelvis	5.5	R_0_ resection	No	96	Alive
10	M	68	Abdominal pain	Pelvis	2.5	R_0_ resection	No	126	Alive

SFT, solitary fibrous tumor; M, male; F, female.

**Table II:** Histopathological findings from the 10 patients.

Marker	1	2	3	4	5	6	7	8	9	10
CD34	+	+	+	+	+	+	+	−	+	+
Bcl-2	−	+	−	+	−	+	−	+	−	+
CD99	+	+	+	−	+	−	−	+	+	−
Vimentin	+	−	−	+	+	+	+	−	+	+
Smooth muscle actin	−	−	−	+	−	+	−	+	+	−
CD117	−	−	−	−	−	−	−	−	−	−
Cytokeratin pan	−	−	−	−	−	−	−	−	−	−
Desmin	−	−	−	−	−	−	−	−	−	−
Epithelial membrane antigen	−	+	+	−	−	−	−	−	−	−
S-100	−	−	+	−	−	−	−	−	−	−
Insulin receptor	+	+	−	+	+	+	+	+	−	−
IGF-1R	+	−	−	+	−	−	−	−	−	−
Ki-67 labeling index, %	>5	2	1	1	5	0	1	2	0	1
Mitotic fields/10 HPFs	10	1	0	1	3	0	1	2	0	0
Cellularity	+	−	−	−	+	−	+	−	−	−
Pleomorphism	+	−	−	−	+	+	+	+	−	−
Necrosis	+	+	−	−	+	−	−	−	−	−
Risk score^a^	5	4	2	4	3	4	4	4	2	1

a Risk model proposed by Demicco *et al* (7): Low risk, 0–2; moderate risk, 3–4; and high risk, 5–6.

CD, cluster of differentiation; IGF-1R, insulin-like growth factor 1 receptor; HPF, high-power field.
